# Harm reduction in hospitals

**DOI:** 10.1186/s12954-017-0163-0

**Published:** 2017-06-05

**Authors:** Malika Sharma, Wiplove Lamba, Alexander Cauderella, Timothy H. Guimond, Ahmed M. Bayoumi

**Affiliations:** 1 Maple Leaf Medical Clinic, Toronto, ON Canada; 2 Division of Infectious Diseases, St. Michael’s Hospital, Toronto, ON Canada; 3 Mental Health and Addictions service, St. Michael’s Hospital, Toronto, Ontario Canada; 4 Centre for Addiction and Mental Health, Toronto, ON Canada; 5 Department of Psychiatry, University of Toronto, Toronto, ON Canada; 6 Centre for Urban Health Solutions, Li Ka Shing Knowledge Institute, St. Michael’s Hospital, Toronto, ON Canada; 7 Department of Medicine, University of Toronto, Toronto, ON Canada; 8 Institute of Health Policy, Management, and Evaluation, University of Toronto, Toronto, ON Canada; 9 Division of General Internal Medicine, St. Michael’s Hospital, Toronto, ON Canada

**Keywords:** Harm reduction, Substance misuse, Opioid-related disorders, People who use drugs, Medication-assisted therapy, Hospital

## Abstract

Despite the high rates of hospitalization among people who use drugs (PWUD), harm reduction interventions have not been widely adopted in inpatient settings. We list several harm reduction practices that we believe should be considered in hospitals. Interventions to decrease stigma, including guidance regarding language and partnering with people with lived experience of drug use, can be implemented expeditiously. Hospitals with a high prevalence of drug use can establish addiction consultation services to address issues including initiation of medication-assisted therapy. Prescription opioids as a treatment for opioid addiction for select patients require further implementation science research to determine how to adapt this intervention for inpatient settings. While the evidence base for needle and syringe programs in the community is strong, implementation science research is required to address how best to integrate such programs in hospitals. Such research is also required to determine the optimal programs to ensure continuity of care post-discharge and retention in addiction-related care. We believe that new evidence generation is required to address the optimal use of peripherally inserted central venous catheters, to determine the relative benefits and harms of treatment contracts for inpatients, and to assess the efficacy of supervised injection services for inpatients. The need for harm reduction programs in hospitals emphasizes the need for a pragmatic, patient-centered, non-judgmental approach to the care of PWUD.

Few publications have addressed how to integrate harm reduction approaches into the care of hospitalized PWUD. Such questions are compelling given the marked increase in opioid use in North America, the high rates of hospital admissions among PWUD, and the strong evidence and increasing adoption of harm reduction interventions in community settings. To date, the literature on care for PWUD in hospitals has focused on recognition and management of medical complications of drug use and overdose, withdrawal, pain management, and initiation of medication-assisted therapy (MAT) [1]. In many hospital settings, these interventions may be incompletely implemented while other harm reduction interventions are rarely used, leading to poor quality care for PWUD, non-adherence to management plans, and early discharge against medical advice.

In this commentary, we outline several harm reduction practices that we believe should be considered by hospital-based practitioners. We classify such interventions into three categories. First, we list interventions that should be implemented immediately because of a strong evidence base or compelling ethical arguments. Second, we list interventions for which there is a solid evidence base in community settings but insufficient evidence regarding implementation in hospitals; such interventions require an implementation science approach to identify methods to optimally introduce these interventions into inpatient settings [2]. Third, we list interventions for which there is sufficient uncertainty or controversy such that additional clinical research is needed prior to implementation.

### Interventions to be implemented immediately

#### De-stigmatizing interventions

Drug use is among the most stigmatized conditions in health care [3]. De-stigmatizing interventions starts with educational programs for all health care providers. Lists of terms to avoid and acceptable substitutes can be easily provided to hospital personnel [3]. Involving people with lived experience of drug use as partners in clinical care delivery and hospital policy committees can help more fully address discrimination and stigma.

#### Establishment of addiction consultation services

Timely referral to inpatient addiction consultation services can assist in prescribing MAT and managing pain in hospitalized PWUD. Initiation of buprenorphine in hospitals is associated with increased entry and sustained engagement in outpatient MAT and decreased illicit opioid use 6 months after hospitalization [4]. While buprenorphine may have less abuse potential and lower overdose risk than methadone, it also blocks the effects of other opioids, even at low doses. Accordingly, methadone may be more effective for patients who continue using ongoing non-prescribed opioids to manage cravings, withdrawal and pain in hospitals. Admitted PWUD, who often have high tolerance to opioids, may be denied prescribed opioids due to safety concerns. However, this may lead to under-treatment of pain, withdrawal, and discharge against medical advice. Addiction consultants can advise about appropriate opioid dosing and non-opioid alternatives.

Addiction services can also provide consultation regarding evidence-based behavioural therapies, mental health and psychosocial interventions to promote coping skills, adherence to medical interventions, early recognition of withdrawal, and patient-centered education on safer drug use practices and can contribute to reducing stigma by educating colleagues and modeling non-judgmental and de-stigmatizing care. Furthermore, fully resourced addiction consultation services can assist in appropriate and consistent referral to outpatient services, including addiction therapy, community harm reduction interventions, provision of naloxone for prevention of overdose, and services to address broader social determinants of health, such as housing, social services, and employment support.

### Interventions requiring implementation science research for hospital settings

#### Prescription opioids

About 10 to 20% of people who use injection drugs do not respond to standard MAT. Six randomized controlled trials have demonstrated that injectable diacetylmorphine (heroin) given under medical supervision is superior to methadone for retaining such patients in addiction treatment and decreasing the use of non-prescribed heroin [5]. Furthermore, a recent non-inferiority study demonstrated that injection hydromorphone had similar results to diacetylmorphine [6]. These studies have been conducted over several months and in outpatient settings. However, hospitals offer opportunities to similarly intervene with well-defined selection criteria, close observation and structured dosing regimens, through medical and social support from addiction specialists. We believe that the existing evidence is sufficiently robust and generalizable to consider prescribing oral and potentially intravenous opioids to inpatients who have not responded to conventional MAT. Additional research is required to assess how to effectively implement and integrate such programs into clinical care and how to transition PWUD who are prescribed opioids into outpatient programs.

#### Provision of sterile drug use equipment

Needle and syringe programs (NSP), which distribute or exchange needles, syringes, and occasionally other sterile equipment to PWUD, have been widely adopted internationally but remain controversial in some parts of the USA. NSP use leads to less risky injection, such as the borrowing and reusing of needles, and reduces the risk of HIV seroconversion [7]. Because some patients will continue to inject drugs while in a hospital, we believe that it is reasonable to provide sterile needles and syringes and disposal kits to inpatients to mitigate the risk of acquiring bloodborne infections. Implementation science approaches are important to determine how NSP can be effectively integrated in ways that minimize associated stigma (e.g. nurse-led exchange, a central location for exchange, or peer-led approaches). Implementation science research can also study how to address attitudinal challenges and safety concerns of hospital staff and fellow patients, how to best educate patients and staff about safely handling and disposing of used supplies and how to integrate provision of sterile equipment into routine discharge procedures. Discharge planning should include education and information on where to obtain such equipment on an ongoing basis.

#### Improving care retention and continuity at discharge

Patient discharge may have special challenges, given that patients may be homeless or unstably housed and many may have few social supports. We believe that program evaluation techniques will be helpful to understand how to facilitate the transition from inpatient care to outpatient management for both health issues related to addiction and those related to other physical and mental health concerns. We believe such evaluations should address which aspects of discharge planning and care coordination may be generalizable across settings, such as outpatient intravenous therapy clinics or mental health follow-up, and which require careful attention to local contexts and circumstances to ensure continuity, such as hiring patient navigators and community workers.

PWUD frequently leave hospital early and against medical advice (AMA), in part due to discrimination from healthcare professionals, cravings or withdrawal. Because of the stigma associated with drug use, the reasons for discharge may not always be apparent, which may lead to conflict. The lack of standards and protocols regarding AMA discharges can lead to variability in practice and poor quality follow-up after discharge. AMA discharge guidelines might address how to safely discharge PWUD leaving AMA, including how to arrange outpatient follow-up, how to access community harm reduction services, and how to manage intravenous lines that are in place at discharge, including options to remove an intravenous line with no questions asked upon request of the patient.

### Future directions for research

#### Optimal use of peripherally inserted central venous catheters PICC

Many PWUD present to a hospital with conditions that are optimally managed with prolonged antibiotics delivered through peripherally inserted central venous catheters (PICCs), such as right-sided infective endocarditis or osteomyelitis. Some practitioners may favour treating people who inject drugs and who have such infections with prolonged oral, rather than intravenous antibiotics, citing concerns about line infections or a possible increased risk of endocarditis given the central location of the line. However, the evidence for the efficacy and safety of oral antibiotics for such infections is weak [8]. In contrast, one small prospective observational study suggested that among carefully selected PWUD who receive a PICC alongside a patient contract and anti-tampering mechanisms safely completed their antibiotic therapy and rates of injecting into PICCs were low [9]. We advocate for PICC lines when clinically indicated for patients in conjunction with comprehensive education as to the potential risks. Nonetheless, there is sufficient equipoise to justify a well-powered randomized controlled trial to evaluate the relative safety of oral antibiotics compared to that of PICC lines among PWUD who need prolonged antibiotic therapy, including outcomes related to both the primary infection and potential complications associated with injecting through a PICC. Components of this trial could also investigate whether methods such as monitoring and tamper-resistant mechanisms are helpful in improving adherence to PICC protocols or detrimental by engendering mistrust leading to non-retention in care.

### Treatment contracts

Patient contracts that define acceptable boundaries of behaviour are widely used but have very little evidentiary support. Physicians are often ethically or logistically unable to enforce contracts or are unwilling to follow through with their threats to expel patients for what is deemed a bad behaviour. Further, contracts can detract from building trust in a clinical relationship when experienced as paternalistic and legalistic and may contribute to stigmatization. Contracts may also be harmful if they facilitate forced addiction treatment of patients who are not ready for such interventions. However, clinicians may feel that a contract is the only way to manage the relationship with a patient who is putting themselves, other patients, or staff at risk for harm. Alternatives may include facilitated dialogue with patients, perhaps involving professional mediators and peers. Given that contracts seem to be commonly used in many hospital settings, we believe that a pragmatic randomized controlled trial comparing contracts to non-contract approaches is warranted before continued use.

### Inpatient supervised injection services

Supervised injection in outpatient settings is associated with decreases in needle sharing, rates of overdose in surrounding neighborhoods, and public drug use and also with increased referrals to addiction treatment and social services [10]. Because many patients will continue to inject while in hospital and such injections can pose risks to both patients and others, research is needed on both the potential benefits and harms of supervised injection services in inpatient settings. Given the complex legal, cultural, economic, and epidemiological questions, a mixed qualitative and quantitative approach is important to fully explore such issues. Additional research might address supervised consumption of drugs through routes other than injection, such as oral ingestion, inhalation, or smoking. However, the evidence base for supervised non-injection consumption is weak. Furthermore, supervised smoking may be particularly challenging in hospitals as it will likely require special inhalation rooms with separate ventilation systems, given hospital smoking prohibitions.

## Conclusion

Our list of possible harm reduction interventions is not meant to be comprehensive; rather, it is based on our collective experience as hospital-based internists, psychiatrists, and addiction specialists. While we have restricted our list to issues related to illegal drug use and focused on opioids, some of our questions are relevant to the management of patients with an addiction to cocaine (such as provision of smoking kits), prescription drugs, or alcohol (for example, the benefits of managed alcohol programs which provide patients with safe, controlled alcohol in-hospital). Because many of our recommendations may be viewed as controversial, engaging in dialogue with hospital-based practitioners and administrators may be required to change the inpatient care culture to one that is more open to harm reduction interventions. Most importantly, our approach highlights the need for a pragmatic, patient-centered, non-judgmental approach to the care of PWUD.
